# Brazilian consensus for diagnosis, management and treatment of hereditary transthyretin amyloidosis with peripheral neuropathy: second edition

**DOI:** 10.1055/s-0043-1764412

**Published:** 2023-04-14

**Authors:** Marcus Vinicius Pinto, Marcondes Cavalcante França, Marcus Vinicius Magno Gonçalves, Marcela Câmara Machado-Costa, Marcos Raimundo Gomes de Freitas, Francisco de Assis Aquino Gondim, Carlo Domenico Marrone, Alberto Rolim Muro Martinez, Carolina Lavigne Moreira, Osvaldo J. M. Nascimento, Anna Paula Paranhos Covaleski, Acary Souza Bulle de Oliveira, Camila Castelo Branco Pupe, Marcia Maria Jardim Rodrigues, Francisco Tellechea Rotta, Rosana Herminia Scola, Wilson Marques, Márcia Waddington-Cruz

**Affiliations:** 1 Universidade Federal do Rio de Janeiro, Hospital Universitário Clementino Fraga Filho, Centro de Estudos em Paramiloidose Antônio Rodrigues de Mello, Rio de Janeiro RJ, Brazil.; 2 Mayo Clinic, Department of Neurology, Rochester, Minnesota, United States.; 3 Universidade de Campinas, Faculdade de Ciências Médicas, Campinas SP, Brazil.; 4 Universidade Univalle, Joinville SC, Brazil.; 5 Escola Bahiana de Medicina e Saúde Pública, Salvador, BA, Brazil.; 6 Universidade Federal do Ceará, Faculdade de Medicina, Fortaleza CE, Brazil.; 7 Pontifícia Universidade Católica do Rio Grande do Sul, Hospital São Lucas, Clínica Marrone e Ambulatório de Doenças Neuromusculare, Porto Alegre RS, Brazil.; 8 Universidade de São Paulo, Faculdade de Medicina de Ribeirão Preto, Ribeirão Preto SP, Brazil.; 9 Universidade Federal Fluminense, Niterói RJ, Brazil.; 10 Universidade Federal de Pernambuco, Hospital das Clínicas, Recife PE, Brazil.; 11 Universidade Federal de São Paulo, Escola Paulista de Medicina, São Paulo SP, Brazil.; 12 Fundação Oswaldo Cruz, Rio de Janeiro RJ, Brazil.; 13 Hospital Moinhos de Vento, Porto Alegre RS, Brazil.; 14 Santa Casa de Misericórdia de Porto Alegre, Porto Alegre RS, Brazil.; 15 Universidade Federal do Paraná, Faculdade de Medicina, Curitiba PR, Brazil.

**Keywords:** Amyloidosis, Peripheral Nervous System Diseases, Amyloid Neuropathies, Familial, Amiloidoses, Doenças do Sistema Nervoso Periférico, Neuropatias Amiloides Familiares

## Abstract

Hereditary transthyretin amyloidosis with peripheral neuropathy (ATTRv-PN) is an autosomal dominant inherited sensorimotor and autonomic polyneuropathy with over 130 pathogenic variants identified in the *TTR* gene. Hereditary transthyretin amyloidosis with peripheral neuropathy is a disabling, progressive and life-threatening genetic condition that leads to death in ∼ 10 years if untreated. The prospects for ATTRv-PN have changed in the last decades, as it has become a treatable neuropathy. In addition to liver transplantation, initiated in 1990, there are now at least 3 drugs approved in many countries, including Brazil, and many more are being developed. The first Brazilian consensus on ATTRv-PN was held in the city of Fortaleza, Brazil, in June 2017. Given the new advances in the area over the last 5 years, the Peripheral Neuropathy Scientific Department of the Brazilian Academy of Neurology organized a second edition of the consensus. Each panelist was responsible for reviewing the literature and updating a section of the previous paper. Thereafter, the 18 panelists got together virtually after careful review of the draft, discussed each section of the text, and reached a consensus for the final version of the manuscript.

## Resumo

Polineuropatia amiloidótica familiar associada a transtirretina (ATTRv-PN) é uma polineuropatia sensitivo-motora e autonômica hereditária autossômica dominante com mais de 130 variantes patogênicas já identificadas no gene *TTR* . A ATTRv-PN é uma condição genética debilitante, progressiva e que ameaça a vida, levando à morte em ∼ 10 anos se não for tratada. Nas últimas décadas, a ATTRv-PN se tornou uma neuropatia tratável. Além do transplante de fígado, iniciado em 1990, temos agora 3 medicamentos modificadores de doença aprovados em muitos países, incluindo o Brasil, e muitas outras medicações estão em desenvolvimento. O primeiro consenso brasileiro em ATTRv-PN foi realizado em Fortaleza em junho de 2017. Devido aos novos avanços nesta área nos últimos 5 anos, o Departamento Científico de Neuropatias Periféricas da Academia Brasileira de Neurologia organizou uma segunda edição do consenso. Cada panelista ficou responsável por rever a literatura e atualizar uma parte do manuscrito. Finalmente, os 18 panelistas se reuniram virtualmente após revisão da primeira versão, discutiram cada parte do artigo e chegaram a um consenso sobre a versão final do manuscrito.

## INTRODUCTION

Amyloidosis is a systemic disorder characterized by extracellular deposition of a protein-derived material, known as amyloid, in multiple organs. It occurs when native or mutant polypeptides misfold and aggregate as fibrils. The amyloid deposits cause local damage to the cells around which they are deposited leading to a variety of clinical manifestations. There are at least 36 different proteins associated with amyloidosis. The most well-known type is associated with a hematological disorder, in which amyloid fibrils are derived from monoclonal immunoglobulin light-chains (AL amyloidosis). This is associated with a clonal plasma cell disorder, closely related to and not uncommonly coexisting with multiple myeloma. Chronic inflammatory conditions such as rheumatoid arthritis or chronic infections such as bronchiectasis are associated with chronically elevated levels of the inflammatory protein serum amyloid A, which may misfold and cause AA amyloidosis. 1

The hereditary forms of amyloidosis are autosomal dominant diseases characterized by deposition of variant proteins, in distinctive tissues. The most common hereditary form is transthyretin amyloidosis (ATTRv) caused by the misfolding of protein monomers derived from the tetrameric protein transthyretin (TTR). Closely related is wild-type TTR (ATTRw), in which the native TTR protein, particularly in the elderly, can destabilize and reaggregate causing nonfamilial cases of TTR amyloidosis. Other proteins that have been associated with forms of hereditary amyloidosis are Aα-chain and gelsolin. 1

TTR is an abbreviation for the name of a protein called transthyretin (Trans-thy-retin), a 127 amino acid protein, which is primarily made in the liver and secreted into the blood in healthy people. In its native state, TTR is a tetramer that transports the thyroid hormone thyroxin and vitamin A (retinol) in the blood. According to the new nomenclature criteria, 2 the recommended pattern to identify the disorders associated to mutations in the *TTR* gene (hereditary ATTR) is ATTRV, where *A* stands for amyloidosis, *TTR* stands for transthyretin and *v* stands for variant or mutant, followed by the clinical manifestation: ATTRv with peripheral neuropathy (ATTRv-PN), ATTRv with cardiomyopathy (ATTRv-CA), etc.

Transthyretin amyloidosis is caused by deposition of TTR amyloid fibrils in various tissues; ATTRv is caused by autosomal dominant mutations in the TTR gene, while ATTRwt stands for wild type ATTR. 3

Transthyretin amyloidosis with peripheral neuropathy, also called transthyretin-related hereditary amyloidosis with peripheral neuropathy, familial amyloid polyneuropathy or Corino de Andrade disease, is an inherited neuropathy, with > 130 pathological variants identified in the TTR gene. The majority of TTR variants cause a “neuropathic” or a “mixed” phenotype, 4 5 although some variants typically manifest as a predominant or isolated cardiomyopathy. 6

Transthyretin amyloidosis with peripheral neuropathy is a disabling and life-threatening genetic condition that leads to death in ∼ 10 years if untreated. The prospects for ATTRv-PN have changed in the last decades, as it has become a treatable neuropathy. In addition to liver transplantation, initiated in the 1990s, there are now at least 3 new drugs approved in many countries and many more are being developed. 7 8

The perspectives for ATTRv-PN have changed significantly in the last decades, as it has become a treatable neuropathy. The first disease-modifying treatment was liver transplantation in 1990. 9 Tafamidis, a potent selective TTR stabilizer, was the first drug to show reduction of disease progression. 10 Diflunisal, an old nonsteroidal anti-inflammatory drug, is a non-selective TTR stabilizer and is another therapeutic option (off label). 11 More recently, two gene silencing drugs (inotersen and patisiran) had very favorable results in large international randomized clinical trials. 12 13 Many new drugs are now being tested or developed.

The present study will focus on the most common form of hereditary amyloidosis – ATTRv, with the main purpose of providing a consensus from the Peripheral Neuropathy Scientific Department of the Brazilian Academy of Neurology for the diagnosis, management and treatment of ATTRv-PN.

## METHODS

In June 2017, the first Brazilian consensus for diagnosis, management and treatment of ATTRv-PN was held in Fortaleza, state of Ceará, Brazil, and published in 2018. 14 Since then, new advances have been introduced, imposing the need to review the existing consensus.

As happened before, a group was formed, comprising 18 Brazilian neurologists, who are members of the Peripheral Neuropathy Scientific Department of the Brazilian Academy of Neurology and considered to be representative experts on the subject. Relevant literature on this subject was reviewed by each participant and used for the individual review of the whole text. Each participant was expected to review the text and send a feedback review by e-mail. Thereafter, the 18 panelists got together in a virtual meeting to finalize the document.

## RESULTS

### Epidemiology

Transthyretin amyloidosis with peripheral neuropathy is considered to be endemic in Portugal, Japan, and Sweden, and also probably in Cyprus, Majorca, and Brazil. 15 16 The most common mutation worldwide, especially in endemic regions, is Val30Met (p.Val50Met) (Portugal, Sweden, Cyprus, Majorca, and Brazil) 16 whereas in most parts of the world, cases of ATTRv-PN are mainly sporadic with great genetic heterogeneity, 17 although specific mutations may be relatively prevalent in certain particular areas.

The incidence of ATTRv-PN varies worldwide, with an estimated incidence of 8.7 cases/million persons/year in Portugal 18 and 0.3 cases/million persons/year in the United States. 19 The prevalence in northern Portugal (Póvoa de Varzim and Vila do Conde) is estimated to be 1:1,108 individuals. 20 In endemic areas of northern Sweden, the prevalence of Val30Met mutation is 4%, with a penetrance of only 11% by 50 years of age. 21 In contrast, penetrance is high in Portugal (80% by 50 years of age) 22 and Brazil (83% by 63 years of age) 23 suggesting that ATTRv-PN is a phenotypically and geographically variable disease. 24 The incidence or prevalence of ATTRv-PN in Brazil is still unknown, but it is estimated that Brazil has > 5,000 cases 25 and although the Val30Met variant is largely the most frequent mutation, there is some genetic heterogeneity. 26

### Pathophysiology

Transthyretin is synthesized in the liver (98%), the choroid plexus, and retina pigmented epithelium. Amyloidogenic mutations destabilize the tertiary and quaternary structure of TTR, causing thermodynamic instability and inducing conformational changes. The dissociation of TTR tetramers into monomers, followed by monomer misfolding, produces fibrils that aggregate and deposit on tissues as amyloid. 3 Autopsy studies found TTR amyloid deposited in almost every tissue, but the most affected are peripheral nerves, the heart, the gastrointestinal tract, the kidneys, the eyes, and the central nervous system. 27 28 The TTR amyloid deposit causes tissue damage by direct compression, obstruction, local blood circulation failure and enhanced oxidative stress. In the peripheral nerves, the disease affects first autonomic and small sensory fibers, causing axonal degeneration, following involvement of the large sensory and motor fibers. 29

### Genetic aspects

The TTR gene contains four exons and is located in chromosome 18. More than 130 pathogenic mutations, which segregate by an autosomal dominant manner, have been described. 30 These mutations are mostly point mutations (missense) and a specific variant (Thr119Met) in individuals that carry the Val30Met variant seems to provide a protective outcome regarding the amyloidogenic potential. 31

The penetrance of ATTRv-PN is incomplete and it seems to be higher in the maternal inheritance. 32 Considering the global distribution of ATTRv, the Val30Met (an amino acid substitution – valine to methionine – in the position 30 of the TTR protein), is the most prevalent mutation worldwide followed by the Ser77Tyr variant. 5 17 25 33 34 This mutation (Val30Met) leads to the classical phenotype dominated by neurological features and is localized closely to the 5'UTR of the *TTR* gene, whereas variants placed in the 3'UTR extremity, such as the Val122Ile, are characterized by the cardiac manifestations as the leading clinical feature. 35 In Brazil, the Val30Met mutation answers for the majority of the ATTRv-PN followed by the Val122Ile variant. 26 36 Probably, there is significant variability around the country and unpublished data suggests that the Val122Ile may be highly prevalent in some regions. Recognizing a patient ancestry is relevant as it may provide a clue to the specific pathogenic variant: the Val30Met is more frequently originated from Portugal or Sweden while the Val122Ile is originated from West Africa. 5 34 35 37

### Clinical characteristics of ATTRv-PN

#### Age at onset

Disease onset of ATTRv amyloidosis varies from the 2 ^nd^ to the 9 ^th^ decade of life, with significant variability in different populations. Based on the age of symptom onset, ATTRv amyloidosis patients can be divided into early onset (< 50 years old) and late onset (≥ 50 years old). In endemic countries, excluding Sweden, the majority of patients have an early onset, with a mean age of onset between 30 and 33 years old. 33 38 39 40 41 In nonendemic regions, late-onset patients predominate, and most of them have a non-Val30Met mutation and no family history of ATTRV amyloidosis. 42 43 44 45 46

#### Sensorimotor and autonomic features

Since the original description by Corino de Andrade, ATTRv-PN has been known as a length-dependent polyneuropathy with a predilection for involvement of small sensory and autonomic fibers. 47 The disease usually starts with pain and paresthesias in the feet, associated to distal lower limb pain and thermal sensory loss followed by light touch loss and ankle hypo/areflexia. Other common initial symptoms are weight loss, impotence, diarrhea/constipation, orthostatic intolerance/hypotension, and/or dry eyes and mouth. Usually, patients start with motor symptoms after a 2-year history of sensation loss, and 4 to 5 years after symptom onset, sensory symptoms start in the hands. Amyloid focal deposition at the wrists frequently causes bilateral carpal tunnel syndrome. Untreated cases inexorably progress to severe motor, sensory and autonomic impairment, cachexia, imbalance, gait disturbances and limb ulcerations. 4 38 48

The classical ATTRv-PN phenotype is characterized by a small fiber-predominant neuropathy, with sensory dissociation, early prominent autonomic involvement, and a positive family history. This is the most common phenotype in early-onset patients, especially from Brazil, Portugal, and Japan. 33 38 41 47 Late-onset patients more frequently have an alternative phenotype, characterized by panmodality sensory loss, early motor involvement, mild autonomic features, severe cardiac involvement, and no family history. 27 41 42 49 This latter phenotype predominates in patients from nonendemic areas. 29 44 50 51 Also, ATTRv may have uncommon phenotypes, including ataxic neuropathy, upper-limb predominant multiple mononeuropathies, and motor predominant neuropathy. 51

Coutinho et al., 38 in a classical manuscript, described a large series of ATTRV-PN and classified the disease into three stages. This is known as the Coutinho stages of ATTRv-PN ( Table 1 ). Another classification frequently used is the modified peripheral neuropathy disability score ( Table 1 ). 15

**Table 1:** Coutinho stages of ATTRv-PN and modified Peripheral neuropathy disability score (mPND)

Coutinho stages	mPND
I. Sensory and motor neuropathy limited to the lower limbs. Mild motor impairment. Ambulation without any gait aids.	I. Sensory disturbances but preserved walking capacity (no motor impairment)
	II. Difficulty walking but no need for a gait aid
II. Gait aid required. Neuropathy progress to upper limbs and trunk. Amyotrophy in upper and lower limbs. Moderate motor impairment.	IIIa. One stick or one crutch required for walking
	IIIb. Two sticks, two crutches or a walker required for walking
III. Terminal stage, bedridden or wheelchair bound. Severe sensory, motor and autonomic neuropathy in all limbs.	IV. Patient confined to a wheelchair or bed

#### Cardiomyopathy

Cardiomyopathy occurs in the late stages of early-onset Val30Met patients but can occur in early stages of late-onset Val30Met and several non-Val30Met mutations. Hereditary ATTR with predominant cardiac involvement is called ATTRv-CA. 52 53 The main features of ATTRv-CA are bundle branch, atrioventricular, and/or sinoatrial blocks, as well as increased thickness of ventricular walls, especially the interventricular septum. 54 The accumulation of amyloid in the heart can lead to restrictive cardiomyopathy, right-sided heart failure, or heart failure with preserved ejection fraction. Electrocardiographic abnormalities include disproportionately low QRS voltage and early conduction system disease. 55 Most patients need a pacemaker during the course of the disease. 54 In the Brazilian population, the most common cardiac abnormalities are nonspecific ventricular repolarization changes, ventricular conduction disturbances, atrial tachycardia, valve thickening, and increased myocardial echogenicity. 56 Bone scintigraphy (PYP and DPD tracers) is highly sensitive and specific for ATTR cardiomyopathy. In the absence of a monoclonal gammopathy, grade 2 or 3 cardiac uptake on bone scintigraphy is essentially diagnostic of ATTR-CA. 57 However, it does not differentiate ATTRv-CA from ATTRwt-CA. Recently the Brazilian Society of Cardiology published a useful position statement on diagnosis and treatment of cardiac amyloidosis. 58

### Myopathy

Myopathy is a rare manifestation of ATTRv. 59 60 It is always accompanied by peripheral neuropathy or cardiomyopathy. Creatine phosphokinase (CPK) is usually normal and the pattern of weakness is proximal and symmetric lower limb predominant weakness. 61 Hereditary ATTR patients with nerve and muscle involvement can present with distal weakness and sensory deficits from the peripheral neuropathy and proximal weakness from the myopathy, mimicking chronic inflammatory demyelinating polyradiculoneuropathy (CIDP).

### Eyes

Vitreous opacity, glaucoma, ocular amyloid angiopathy and dry eyes are common and occur in most of the patients during the disease. 62 The full spectrum of the ophthalmological manifestations associated to ATTR have been recently reviewed. 63

### Renal

Renal disturbances are variable in ATTRv-PN, and proteinuria seems to be the first finding. Patients can progress to nephritic or nephrotic syndrome and renal failure. It is estimated that one third of Portuguese ATTRv-PN patients develop nephrotic syndrome and renal failure. 64 Recently, it was shown that Tafamidis dramatically improved a severe proteinuria present in a patient with the Val30Met variant. 65

### Central nervous system

Central nervous system symptoms are a common late complication in Val30Met ATTRv-PN patients after 15 years of symptomatic disease. 66 Transient focal neurologic episodes (positive: visual hallucinations, tingling, motor activity; negative: aphasia, visual loss, hemiparesis), intracerebral hemorrhage, ischemic strokes, and cognitive decline can occur secondary to amyloid deposition in the meningeal vessels of the brain and brainstem. These amyloid fibrils are formed mostly by TTR produced in the choroid plexus and are resistant to available ATTRv disease-modifying therapies. 67

Patients with ATTRv with non-Val30Met mutations can also present with a rare phenotype of oculoleptomeningeal amyloidosis. These patients present early in their disease course with prominent ocular and CNS symptoms. Fourteen mutations have been described with this phenotype. 67 Recently, one patient with Tyr69His ATTRv oculoleptomeningeal amyloidosis was reported here in Brazil. 68

## DIAGNOSIS

### Symptoms and signs

The clinical picture of ATTRv-PN is not exclusive. It is very important for the clinician to know the red flags for suspecting ATTRv-PN, consider genetic testing and, in some cases, biopsy. In patients with progressive undetermined sensorimotor polyneuropathy, one or more of the following features should raise the suspicion of ATTRv-PN 7 69 :

- Family history of neuropathy;
- Orthostatic hypotension;
- Sexual dysfunction (erectile dysfunction);
- Unexplained weight loss;
- Arrhythmias, conduction blocks, cardiac hypertrophy and cardiomyopathy;
- Bilateral carpal tunnel syndrome;
- Renal abnormalities (proteinuria or azotemia);
- Vitreous opacities;
- Gastrointestinal complaints (chronic diarrhea, constipation or diarrhea/constipation, early satiety);
- Rapid progression; and
- Prior treatment failure.

Whenever ATTRv-PN is suspected on clinical grounds, one should move forward and order *TTR* gene sequencing to confirm the genetic diagnosis. In some patients, pathological evidence of amyloid deposits is also recommended in the diagnostic work-up. 70

### Tissue biopsy

**Figure 1:**
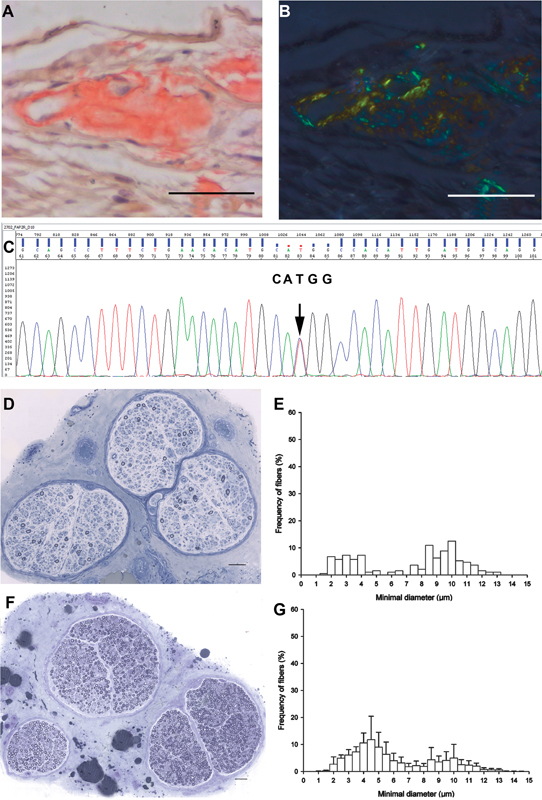
A. Amyloid material deposition in a vessel wall (left) and in the adjacent endoneurial space on Congo red staining (sural nerve biopsy). B. The section A under polarized light shows the amyloid material birefringence appearing here as apple-green and golden-yellow colors. C. Electropherogram of TTR gene shows the c.148G > A(Val30Met) mutation. D. Semithin section stained with Toluidine Blue shows axonal loss. F. Normal sural nerve for comparison with D. E. Percentage histograms of the myelinic fibers seen in D demonstrate the predominance of thin myelinated fiber (7 µm or less diameter) loss in comparison with the normal histogram represented in G. Scale bars = 50 µm. Images A-E are from the same patient specimens.

Confirmation of amyloid deposition via tissue biopsy is recommended but not mandatory. The labial salivary gland, peripheral nerve biopsies and fat pad aspirate are usually the sites of choice. Other tissues can be biopsied, like rectum, carpal flexor retinaculum, skeletal muscle, skin or endo/myocardium. 15 In Brazil, the preferred sites are the labial salivary gland and peripheral nerve ( Figure 1 ). 14 It is important to note that a negative biopsy does not exclude the diagnosis of ATTRv-PN. If the suspicion is still high, another tissue biopsy and genotyping need to be planned. On peripheral nerve biopsy, amyloid deposits are scattered in the endoneurium and around blood vessels and have a round, amorphous, and orange appearance on Congo red staining, with characteristic apple-green birefringence under polarized light 61 71 ( Figure 1 ). The sensitivity of labial salivary gland biopsy in Val30Met ATTRv-PN patients is high, and varies between 75 and 91%. 72 73 Skin biopsy sensitivity varies between 70 and 80% 61 . Fat pad aspirate sensitivity for ATTRv is ∼ 45%. 74 Small studies suggest that nerve biopsy sensitivity for detection of amyloid deposits can be very high with serial sections of the whole nerve specimen (up to 93%). 5 75

Immunohistochemistry can identify whether the amyloid deposit comprises of TTR, but it does not differentiate mutated from wild-type TTR. Mass spectrometry-based proteomics of the amyloid deposit can identify the misfolded protein, and even differentiate mutated from wild-type TTR. 29

### Genetic test

The final diagnosis of ATTRv-PN relies upon the identification of a pathogenic TTR variant. Whenever possible, the sequencing of all exons and exon-intron boundaries of the TTR gene should be obtained. 70 This is particularly important for patients with no obvious family history. 15 70 Sequencing can be accomplished either by Sanger or next-generation sequencing (NGS) pipelines. In families with a known mutation, direct investigation of the specific variant can be performed in relatives. It is important to note that whole-exome or whole-genome sequencing can provide false negative results.

Presymptomatic testing may be done in at-risk persons. It is essential that this procedure be performed after the patient has expressed a favorable response and that it is preceded by pertinent genetic counseling; ideally, under the command of a geneticist or neurogeneticist. 76

### Differential diagnosis

Toxic, metabolic, inflammatory, infectious, and other inherited neuropathies must be ruled out. According to some studies, CIDP is the most common misdiagnosis, especially in late-onset patients without a family history. Cortese et al. 77 showed that from 150 patients, 32% had been misdiagnosed and 61% were thought to have CIDP. One important rule is to consider the diagnosis of ATTRv-PN in a CIDP patient that does not respond to immunomodulatory and/or imunossupressor treatments. 69 Amyloidosis may have patchy deposition, then could be misdiagnosed as radiculopathy or plexopathy. 78 ATTRv-PN rarely causes proximal and distal weakness, which is very common in CIDP, and seldom fulfills the European Federation of Neurological Societies/Peripheral Nerve Society nerve conduction criteria for CIDP. 77 79

Immunoglobulin light chain amyloidosis is another important differential of ATTRv-PN. Serum and urine immunofixation help to differentiate these disorders, but ATTRv-PN patients may also have monoclonal gammopathy of uncertain significance and this is not unusual in late-onset cases. 61 Mass spectometry-based proteomics of the amyloid deposit can differentiate which type of misfolded protein is deposited on tissues, and DNA analyses should always be requested in ATTRv-PN-suspected cases ( Table 2 ). 15

**Table 2:** Differential diagnosis of hereditary transthyretin amyloidosis with peripheral neuropathy

Differential diagnosis	Clues for the differential diagnosis
Diabetic neuropathy	Poor glycemic control and mild motor involvement
CIDP	Proximal and distal weakness and non-uniform demyelination on NCS
Leprosy	Multiple mononeuropathies/asymmetric neuropathy, typical skin lesions
Toxic neuropathies	Bortezomib, thalidomide, vincristine, alcohol abuse
Fabry	Angiokeratomas, stroke, and alpha-glucosidase deficiency
Charcot-Marie-Tooth	Mild sensation loss and no autonomic involvement
HSAN	No or mild motor involvement
Immunoglobulin light-chain amyloidosis	Monoclonal gammopathy in the serum and/or urine, abnormal kappa/lambda ratio, mass-spectrometry, bone marrow biopsy

Abbreviations: CIDP, chronic inflammatory polyradiculopathy; HSAN, hereditary motor and autonomic neuropathy; NCS, nerve conduction studies.

### Management

Transthyretin amyloidosis with peripheral neuropathy is a complex multiorgan disease that requires comprehensive multidisciplinary care. Recent multinational collaborative efforts have attempted to provide international guidelines for early treatment and screening for medical complications in ATTRV-PN patients and stimulated the development of amyloidosis referral center and national and international networks to exchange of experiences and information about new therapies and clinical trials. 76 80 81 It is important to emphasize the need for close follow-up in centers specialized in the management of the different neurological and medical complications experienced by these patients, since early recognition of the different medical complications is of paramount importance. 76 Since a significant number of patients do not have access to ATTRv-PN treatments, clinicians should be aware of the different aspects of medical management. Symptomatic treatment will not be discussed in the present review. The reader is referred to excellent reviews elsewhere. 63 82 83 84 85 Neurologists, cardiologists, internists, nephrologists, ophthalmologists, general practitioners, neurogeneticists, mental health providers, nutritionists, nurses, and physical therapists need to work together to improve patient care and quality of life.

As treatment options increase, monitoring disease progression is becoming more and more important in the follow-up of these patients. The proposed monitoring of neurological aspects includes the possibility of use of several different assessments depending on the availability and experience of the center. 86 The frequency of monitoring needs to be individualized and adapted to the course and to the severity of the disease, in general 6 to 12 months, preferably done by the same evaluator. A direct anamnesis that includes important neuropathic symptoms, autonomic dysfunction complaints, full neurological examination of the four limbs, covering all sensory modalities, comprise the basis of the assessment. Quantitative measures that are commonly used are: neuropathy impairment score (NIS), polyneuropathy disability score (PND), 6-minute walt test (6-MWT) and timed 10-meter walk test (10-MWT). The suggested indicator of progression for NIS is a change of 7 to 16 points or worsening of the score on 2 consecutive consultations 6 months apart, considering more important a change in strength than in reflex, highlighting again the importance of the judgment of the clinician. Routine nerve conduction study, sympathetic skin response, laser evoked potentials, and quantitative sensory test are other proposed tests. Modified NIS + 7 composite clinical and neurophysiologic score is not frequently used outside clinical trials as it is time consuming and not widely available. Other tests, such as postural hypotension, heart rate variability, urodynamic tests, Sudoscan, and Compass 31 QOL questionary offer a measure of autonomic dysfunction. Body mass index (BMI) and modified BMI (mBMI) is largely used for the nutritional status. Functional ability in daily life can be measured by Rash-built overall disability scale (R-ODS). A recent expert consensus proposed a minimal set of evaluation composed of: NIS, PND, 6-MWT or 10-MWT, Compass 31 QOL, and R-ODS done at least once a year. A final decision on disease progression remains the clinician decision after considering individual aspects and test sensitivity for the specific phenotype.

### Disease modifying treatments

**Figure 2:**
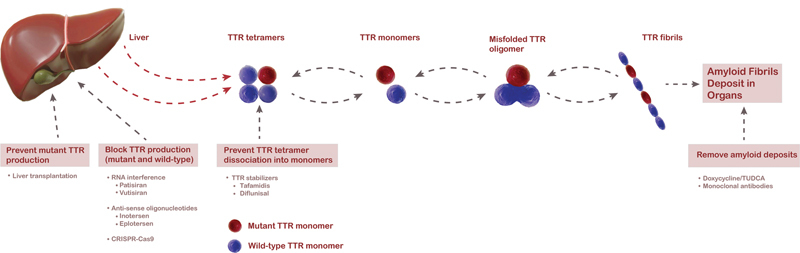
TTR amyloidogenesis and disease modifying therapies.

Disease modifying strategies may target any of the key steps that end with TTR fibril deposition ( Figure 2 ).

#### Liver transplant

Most of the circulating TTR (98%) is produced by the liver. Accordingly, liver transplantation was introduced in order to stop production of the mutated TTR and the consequent amyloid deposition, aiming a potential cure for the disease. 87 The first orthotopic liver transplant in ATTRv-PN was carried out in Stockholm, Sweden, in 1990. 88 In South America, the first liver transplant performed for this purpose was in São Paulo, Brazil, in 1993. 89 The first series of orthotopic liver transplants showed a decrease in the amyloid load and improvement of symptoms in some patients. This suggested that the procedures were successful, and the cure for this fatal disease was finally achieved. 90 However, subsequent studies showed that the results were not good in old patients, and in those who were malnourished and/or had an advanced disease and/or had non-Val30Met mutation. 9 91

In 2015, the Familial Amyloid Polyneuropathy World Transplant Registry published its experience of 2,127 liver transplants in 1,940 ATTRv-PN patients. The overall 20-year survival after transplantation was 55.3%, and independent risk factors for good prognosis were: early-onset, Val30Met mutation, modified BMI before transplant, and a short disease duration. 92 However, a liver transplant does not interfere with eye or central nervous system amyloid deposition, as the retina and the choroid plexus continue secreting mutated TTR. 66 93 Transplantation of livers from ATTRv-PN donors have been considered when the prospective recipients with other liver diseases would otherwise have a long wait or are seeking palliation rather than long-term cure. This is known as a sequential, or domino, liver transplant and has the advantage of addressing organ shortage and allowing transplantation with less ischemic time. Recipients of a domino ATTR liver can develop analogous neurological manifestations as early as several months to years after the transplantation; the symptoms could worsen despite re-transplantation from a healthy donor to replace the first transplanted amyloidogenic liver. Patients also have the option of re-transplantation in the future. 94

After the introduction of alternative therapeutic possibilities, the option for liver transplantation has significantly decreased. 92 Patients with hereditary transthyretin (TTR) amyloidosis (hATTR) often experience disease progression after orthotopic liver transplant (POLT) due in part to wild type ATTR amyloid deposition. In 2020, Moshe-Lilie et al. published a case series of 9 postliver transplanted hATTR patients (8 non V30M) were treated with inotersen based on the fact that TTR silencers suppress both TTRv and TTRwt. From those, neuropathy impairment score remained stable or improved in all patients after 12 months of treatment, but 5 patients stopped treatment: 3 presented thrombocytopenia and 2 presented a reversible liver rejection. *TTR* gene silencing therapy in hATTR patients with POLT can be a treatment option, but close monitoring is needed, because of frequent clinical complications.

### TTR stabilizers

#### Tafamidis

Tafamidis binds with selectivity, high affinity, and negative cooperativity to wild-type or mutated TTR, increasing TTR stability and impeding TTR dissociation, the rate-limiting step of amyloid formation 95 ( Figure 2 ). A Tafamidis Phase II/III trial, FX-005, evaluated the efficacy and safety of Tafamidis (20 mg once daily) in an 18-month randomized, double-blind, international multicenter, placebo-controlled trial that enrolled 128 patients. 10 The primary outcome measures were the Neuropathy Impairment Score of the Lower Limbs (NIS-LL) and the Norfolk QOL-DN at 18 months. Secondary outcome measures were composite scores of large and small fiber functions and the modified BMI. Primary efficacy endpoints were analyzed in the intention-to-treat (all patients randomized) population and the efficacy-evaluable population (population that completed the study) that was prespecified, assuming a dropout of patients for liver transplant, as many of them were on the transplant waiting list. The greater proportion of patients in the Tafamidis group was NIS-LL responders, who had better quality of life. In the intention-to-treat analysis, the difference was not statistically significant for NIS-LL responders (45.3 versus 29.5%; *p* = 0.068) or for the treatment group differences in the Least-Square Norfolk QOL-DN (- 5.2-point difference; *p* = 0.116). However, the efficacy-evaluable analysis showed significantly more NIS-LL responders (60.0 versus 38.1%; *p* = 0.041) and a significantly better quality of life in the Tafamidis group (Least-Square Norfolk QOL-DN - 8.8-point difference; *p* = 0.045). Analysis of secondary outcome measures showed that Tafamidis reduced the deterioration of neurological functions and improved the nutritional status of the patients. 10

This trial faced a higher than anticipated dropout rate due to liver transplants (21% observed versus 10% estimated), equally distributed in both arms. The choice of patients who underwent a liver transplant as nonresponders influenced the analyses of NIS-LL in the intention-to-treat population, possibly underpowering the effect on the NIS-LL progression. In spite of the limitation to demonstrate statistical significance in primary outcomes, the totality of the results demonstrated the potential of Tafamidis to slow neurologic deterioration and maintain nutritional status. 96

Subsequently, an open label extension study (FX-006) enrolled the remnants of the FX-005 (20 mg/day) showing the benefits of Tafamidis were sustained for 30 months. In addition, those patients who were in the placebo arm at the FX-005 continued to progress faster after starting taking Tafamidis, and initiation of Tafamidis in patients with mild peripheral neuropathy (NIS-LL ≤ 10) provided more sustained benefit, showing that early initiation of Tafamidis was associated with better response and outcome. 97 98 99 Additional studies showed that Tafamidis provided long-term (up to 6 years) delay in neurological deterioration and nutritional status in Val30Met patients. 100

Tafamidis was found to be effective in stabilizing serum TTR in non-Val30Met patients. 101 102 Recently, a large natural history study of Val30Met ATTRv-PN patients from Portugal demonstrated that Tafamidis decreased the mortality risk compared with untreated patients by 91% in early-onset patients and by 82% in late onset patients with this specific mutation. 103 In this study, the 10-year probability of survival for patients on Tafamidis and untreated was 96 and 72%, respectively, in early onset patients, and 92 and 49%, respectively, in late onset patients. 103

There is strong evidence that the drug is safe, has good tolerability and few side effects (diarrhea and urinary infection). Tafamidis was approved by Brazil's Health Agency (ANVISA, in the Portuguese acronym) for the treatment of ATTR-FAP and has been incorporated at our Brazilian unified health system (SUS, in the Portuguese acronym) to treat ATTRV-PN.

Recently, Tafamidis was also found to be effective for the treatment of ATTR-CA at a dose of 80 mg/day, reducing mortality and functional decline, as well as preserving quality of life. 104 105

#### Diflunisal

Diflunisal is an anti-inflammatory non steroid drug (NSAID) developed > 30 years ago that nonselectively stabilizes TTR. 80 The diflunisal Phase II/III trial was a 24-month randomized, double-blind, international multicenter, placebo-controlled trial that enrolled 130 patients with Val30Met and non-Val30Met mutations, in all Coutinho stages. The primary end points were stabilization on the Neuropathy Impairment Score plus seven neurophysiological tests (NIS + 7). After 2 years, diflunisal was shown to reduce disease progression. The dropout rate was 50% in the placebo group and 25% in the treatment group. Most of the patients dropped out because of disease progression, liver transplant, and side effects. 11 Although the study did not show high rates of side effects in the diflunisal group, there is a serious concern about the long-term effects of this NSAID on the kidneys, heart, and gastrointestinal tract. 80 A retrospective analysis of diflunisal off-label use showed that 57% of the patients discontinued therapy, mostly because of gastrointestinal disorders. 106 The Swedish study DFNS01 107 was a 24-month open-label observational study designed to monitor the effect of diflunisal 500 mg daily in ATTRv. Fifty-four patients were included. Seventeen (31%) of the patients had completed the 24- month study follow-up, whereas 37 (69%) had dropped out after a mean duration of 10.8 (0.4–21.8) months. The main reasons for early termination were liver transplantation (24%), and side effects (19%). The most frequent side effects were dyspepsia (12%), diarrhea (9%) and increased of serum creatinine (7%). Motor neuropathy scores and cardiac septum thickness increased significantly during the study, which suggests that complete disease stabilization was not achieved, but the number of patients was low in this study. Also, it is important to note that patients with renal dysfunction were excluded from the diflunisal trial 11 and that diflunisal has not been approved for the treatment of ATTRv-PN by any health agency (off-label use only).

### TTR gene silencing

#### Antisense oligonucleotides

Inotersen is an antisense oligonucleotide inhibitor that binds to TTR messenger RNA (mRNA) impeding transcription by inducing its cleavage ( Figure 2 ). Animal and human studies have shown a robust suppression (> 80%) in TTR serum levels. 108 109 The Phase 3 Study IONIS-TTR Rx was a randomized, double-blind, international multicenter placebo-controlled trial (NEURO-TTR trial), with weekly subcutaneous injections of the study drug in adults with stage 1 or stage 2 ATTRv-PN. 13 Primary endpoints were modified NIS + 7Ionis and Norfolk QOL-DN. A total of 172 patients (112 in the inotersen group and 60 in the placebo group) were included, and 139 (81%) completed the trial. For NIS + 7ionis, the least-squares mean change from baseline to week 66 between the two groups (inotersen minus placebo) was - 19.7 points ( *p* < 0.001) and for the Norfolk QOL-DN it was - 11.7 points ( *p* < 0.001). There were five deaths in the inotersen group and none in the placebo group. The most common serious adverse events in the inotersen group were glomerulonephritis (3 patients) and thrombocytopenia (3 patients), with one death associated with one case of severe thrombocytopenia. The other deaths in the inotersen group were due to cachexia (2), intestinal perforation (1) and congestive heart failure (1). The 2-year open-label extension of the NEURO-TTR trial reassured the long-term benefit in terms of neuropathy progression, neuropathy-related QOL and health-related QOL, with no additional safety concerns. Importantly, this open-label extension showed better outcomes in patients from the inotersen group from the beginning than patients from the placebo group who switched to inotersen in the extension study. Inotersen slowed the course of neurologic disease and improved quality of life in patients with ATTRv-PN, with better results when started early. 110 Inotersen has been approved by ANVISA for the treatment of ATTR-FAP but so far has not been incorporated by the SUS.

Eplotersen is a ligand-conjugated antisense (LICA) drug that shares the same nucleotide sequence as inotersen, but has an advanced design that increases drug potency to allow for lower and less frequent dosing. 111 This LICA is administered subcutaneously every 4 weeks. A phase 3 clinical trial is underway comparing the efficacy and safety of eplotersen versus inotersen (NCT04136184)

#### Small interfering RNAs

Patisiran is a small interfering RNA that binds to specific coding regions of TTR mRNA suppressing TTR production. Preliminary studies showed that patisiran inhibited more than 80% of TTR production. 112 The phase III APOLLO study was a randomized, double-blind, international multicenter placebo-controlled trial, with intravenous infusion of the study drug every 3 weeks at the dose of 0.3 mg/kg. 12 A total of 225 patients were randomized (148 to the patisiran group and 77 to the placebo group). The primary end-point was modified NIS + 7 _Alnylan_ The least-squares mean change from baseline to 18 months between groups (patisiran minus placebo) for NIS + 7 _alnylan_ was - 34.0 ( *p* < 0.001) and for Norfolk QOL-DN was - 22.1 ( *p* < 0.001). Approximately 20% of the patients who received patisiran and 10% of those who received placebo had mild or moderate infusion-related reactions; the frequency and types of adverse events were similar in the two groups. Death occurred in seven patients in the patisiran group and sin ix patients in the placebo group. In this trial, patisiran improved numerous clinical manifestations of ATTR-FAP. The 12 month open label extension trial of Patisiran for ATTRv-PN continued to demonstrate the benefits and safety profile of this RNAi. 113 Also, this study emphasized the importance of early treatment to halt or reverse the progression of the polyneuropathy, malnutrition, and quality of life impairment. 113 Patisiran has been approved by ANVISA for the treatment of ATTR-FAP but so far has not been incorporated at SUS.

Vutrisiran is a subcutaneous small interfering RNA administered every 3 months that utilizes a GalNAc-conjugate delivery platform. 114 It is currently being tested in a multicenter Phase III clinical trial to treat ATTRv-PN neuropathy (NCT03759379).

### Doxycycline and TUDCA

Doxycycline and TUDCA may promote the removal of TTR deposits and repair the remaining tissue. They have a synergistic effect and work by lowering both fibrillar and non-fibrillar amyloid deposits. A Phase II clinical trial showed that this combination stabilizes the disease in patients with ATTRv amyloidosis with good tolerability and few side effects. 115

### Emerging drugs

#### CRISPR-Cas9

A recent study demonstrated that *TTR* gene editing was achieved using a clustered regularly interspaced short palindromic repeats and associated Cas9 endonuclease (CRISPR-Cas9) system in 6 patients with ATTRv-PN. 116 Single doses of the study drug, NTLA-2001, at day 28, was associated with mean TTR reductions of 52% in the group that received a dose of 0.1 mg per kilogram and 87% in the group that received 0.3 mg per kilogram to achieve in vivo gene editing. Patients had only mild reactions to the study drug. 116 This approach has the potential to treat all forms of ATTR amyloidosis — both wild-type and hereditary disease, and as it is only a single dose, treatment compliance would not be an issue. However, larger clinical studies with long follow-up are necessary to confirm If CRISPR-Cas9 will produce a new revolution in the treatment of ATTRv-PN.

#### Monoclonal antibodies

Therapeutic amyloid-directed antibodies that specifically bind, disrupt and remove amyloid deposits are under investigation. PRX004 is an anti-TTR monoclonal antibody that binds to residues 89–97 of the TTR protein. Twenty-one ATTRv patients were enrolled in a phase I open label dose escalation study of PRX004 (dose cohorts: 0,1, 0.3, 1, 3, 10 and 30 mg/kg IV infusion every 28 days for up to 3 infusions), where the drug had an overall safe side effects profile and was well tolerated. Seventeen of these patients were enrolled in a long-term extension study to receive up to 15 infusions of PRX004. At month 9, all the 7 evaluable patients showed improvement/slower progression in neuropathy versus disease natural history. 117 NI301A is a recombinant human monoclonal immunoglobulin G1 that binds selectively with high affinity to the disease-associated ATTR amyloid deposits. In a phase 1 clinical trial in patients with ATTR cardiomyopathy, it was safe and well tolerated. NI301A removes ATTR deposits ex vivo from patient-derived myocardium by macrophages, as well as in vivo from mice grafted with patient-derived ATTR fibrils in a dose- and time-dependent fashion.

### Therapeutic strategy

There is no direct comparison among the three drugs that have been approved by ANVISA. Tafamidis is an oral drug that has been approved to ATTRv-PN stages 1 and 2, but it seems that the earlier it is introduced the best will be the result. 98 99 Monteiro et al. 118 have shown that the neuropathy stabilizes in almost one-third of the patients; another third responds well for a shorter period and the remaining third do not respond at all.

Considering this data, plus the safety profile, the facility of use and the involved costs, we think Tafamidis should be considered as the first option at the early stage of the disease. These patients should be followed-up closely clinically and electrophysiologically and at the first evidence of disease progression a second drug should be introduced.

Both inotersen and patisiran seem effective in controlling the disease. As there is no direct comparison among them, the choice should be directed by availability, ease of use and patient/family preferences. Anyway, these patients should also be followed-up closely and any evidence of an unsatisfactory response should prompt trying the remaining alternatives. It is unclear what is the current role of liver transplantation in this new era of medical therapies for ATTRv-PN.

The expanding treatment options introduced in the clinical practice the necessity to identify for all treatment options the concept of responders and nonresponders. Authors from the Amyloidosis center Corino de Andrade at Hospital Santo Antônio do Porto, together with the Scripps Institute in California, carried out a retrospective analysis of 210 patients with V30M ATTR with predominantly neuropathic phenotype treated by Tafamidis for 18 to 66 months. 118 The aim was to determine long-term effectiveness of Tafamidis in real-life practice and to look for clinical characteristics and plasma biomarkers that could be used as outcome predictors of treatment response. Patients were classified by an expert in responders, partial responders and nonresponders. The expert judgment was based on the review of different aspects of the disease including: neuropathy impairment score (NIS), Norfolk Quality of Life Questionary (Norfolk QOl), measures of routine compound nerve action potentials (neurophysiological score), nutritional status, cardiology, and nephrology visits. Responders corresponded to 34.3% of the patients (no disease progression, NIS change from baseline ≤ 0). Nonresponders (29%) presented worsening of sensory, motor and autonomic neuropathic aspects as expected without any treatment (NIS increase of 5.9/year). Partial responders (36%) were considered based on progression of sensory and/or motor aspects of the neuropathy with significant improvement of autonomic aspects, or continued progression overall but slowly than nonresponders (NIS increase of 1.8 / year). The authors determined that lower disease severity, female sex, and native higher levels of tetrameric TTR concentration at onset of treatment were the most relevant good predictors of response. Plasma levels of Tafamidis at 12 months of therapy was also a predictor of response for male patients. We believe that similar studies should be available to all treatment options.

In conclusion, ATTRv neuropathy is a severe and progressive neuropathy that impairs quality of life and shortens significantly the existence. Early diagnosis and treatment are essential to avoid the natural history of the disease. Once diagnosed, these patients should be followed by a multidisciplinary team with expertise in this disease, in order to offer them the best individualized treatment approach at the proper time.
